# A multinational survey on the infrastructural quality of paediatric intensive care units

**DOI:** 10.1186/s13613-018-0451-1

**Published:** 2018-11-06

**Authors:** Gert Warncke, Florian Hoffmann, Michael Sasse, Georg Singer, Istvan Szilagyi, Holger Till, Christoph Castellani

**Affiliations:** 1 Department of Pediatric and Adolescent Surgery, Medical University of Graz, Auenbruggerplatz 34, 8036 Graz, Austria; 2 Children’s Hospital, Ludwig-Maximilians-University Munich, Lindwurmstrasse 4, 80337 Munich, Germany; 3 Department of Pediatric Cardiology and Intensive Care Medicine, Medical School Hannover, Carl-Neuberg-Strasse 1, 30625 Hannover, Germany

**Keywords:** Paediatric, Intensive care, Quality management, Personnel, Organization, Infrastructure

## Abstract

**Background:**

The aim of the present study was to assess whether paediatric intensive care units (PICUs) in three central European countries comply with guidelines concerning infrastructure provided by the European Society of Intensive Care Medicine (ESICM). Between July 2016 and May 2017, a survey was conducted based on the ESICM guidelines. The questionnaire was structured into four categories: structural quality, diagnostic/therapeutic equipment, personnel and organization. All PICUs treating paediatric patients in the D–A–CH region [Germany (D), Austria (A) and Switzerland (CH)] were researched through the national societies. A total of 126 PICUs were contacted (D: 106; A: 12; and CH: 8).

**Results:**

Eighty-five of 126 PICUs responded (D: 67%; A: 61%; and CH: 100%). A median of 500 patients was treated annually (D: 500; A: 350; and CH: 600) with a median of 12 beds (D: 12; A: 8; and CH: 12). Recommendations regarding infrastructure were met as follows: structural quality 62% in D, 71% in A and 75% in CH; diagnostic/therapeutic equipment: 87% in D, 91% in A and 89% in CH; personnel: 65% in D, 87% in A and 85% in CH; and organization: 75% in D, 73% in A and 88% in CH.

**Conclusions:**

This survey reveals deficits concerning structural quality in all countries. Furthermore, shortcomings regarding personnel were found in Germany and for organization in Germany and Austria. These issues need to be addressed urgently to further improve treatment quality and patient safety in the future.

**Electronic supplementary material:**

The online version of this article (10.1186/s13613-018-0451-1) contains supplementary material, which is available to authorized users.

## Background

Paediatric intensive care represents a highly specialized discipline with a prominent role in the treatment of critically ill children [1–3]. Multiple technical innovations with sophisticated therapeutic and diagnostic possibilities such as extracorporeal membrane oxygenation (ECMO), dialysis or plasmapheresis are not only increasingly used in paediatric intensive care, but also required specially trained operators [4–6]. These refinements coupled with changed regulations concerning working hours in the European Union have led to challenges regarding personnel, organization and economics [7].

All these issues have generated concerns regarding quality management (QM) in paediatric intensive care. Consequently, European countries attempt to legislate QM strategies in patient care aiming to optimize therapy regimes [8]. In this context, intensive care—especially in children—poses an enormous challenge. The patient collective is inhomogeneous with a wide range of physiological and psychological differences. Additionally, paediatric intensive care frequently requires a multidisciplinary approach with cooperations exceeding the treatment of adults [3, 9].

Focusing on paediatric intensive care medicine, QM guidelines have been published by different societies such as the “European Society of Intensive Care Medicine (ESICM)” on the European level and the “German Interdisciplinary Association of Critical Care and Emergency Medicine (DIVI)” on the national level for Germany [10–12]. These guidelines focus—amongst others—on structural quality and their adherence seems to have an indirect influence on the quality of results [7, 13–17]. However, it still remains unclear whether PICUs in D–A–CH countries (Germany, Austria and Switzerland) meet these European recommendations. The aim of this investigation was to assess PICU compliance (D–A–CH) with European guidelines regarding QM infrastructure.

## Methods

This survey was based on the 2011 ESICM guidelines “Recommendations on basic requirements for intensive care units: structural and organizational aspects” [11]. In some aspects, however, the ESICM guidelines remain unspecific and were thus supplemented by the DIVI recommendations [12]. This study focuses on infrastructural quality only (as initially defined in the Donabedian framework) [18]. The infrastructural recommendations were structured into the following four categories: *structural quality*, *diagnostic and therapeutic equipment*, *personnel* and *organization and quality improvement*. Based on these data, a committee of experts in this field a questionnaire focusing on infrastructural quality of paediatric ICUs was generated (Additional file 1).

Country-specific national societies identified those units that were providing PICU care in Germany, Austria and Switzerland. Intensive care units organized as separate neonatal ICU (NICUs) were excluded from this survey. For practical reasons, the term PICUs will refer to all intensive care units contacted (paediatric, paediatric surgical or combinations). Contact data of the heads of the PICUs were obtained from the internet or provided by the national societies. Between July 2016 and May 2017, the questionnaire was sent to a total of 126 PICUs of which 106 were located in Germany (D), 12 in Austria (A) and 8 in Switzerland (CH).

Since no additional interventions were performed, the need for informed consent was waived by the institutional review board.

Data were managed with Microsoft Excel 2011^®^. All statistical analyses were performed using SPSS 21.0^®^. Ordinal and nominal data are presented as number (n) and per cent. The percentage of valid responses was recorded for each item. The results were related to the total amount of valid responses for each item. Web diagrams were used to demonstrate to which percentage PICUs in the different countries complied with the recommendations. Additionally, the following sub-categorizations were performed: (1) university hospital and non-university hospital and (2) ≤ 400 patients treated annually and > 400 patients treated annually.

## Results

### General findings

The overall availability of PICU beds was 0.5/10,000 children (≤ 18 years) in Germany, 0.6/10,000 children in Austria and 0.7/10,000 children in Switzerland. A total of 126 eligible PICUs were contacted. A response could be obtained from 85 of these (Germany 65/106; Austria 12/12; and Switzerland 8/8), resulting in an overall response rate of 67.5%. One German PICU had treated 0 patients in the surveyed period and was thus removed from further analysis.

The median number of annually treated patients was 500 ranging from 20 to 1400 (median for D: 500; for A: 350; and for CH: 600); the detailed data for the different sub-categories are provided in Additional file 2. The median duration of PICU stay was lowest in Switzerland (3 days) followed by Austria (6 days) and Germany (7 days).

### Structural quality

**Fig. 1:**
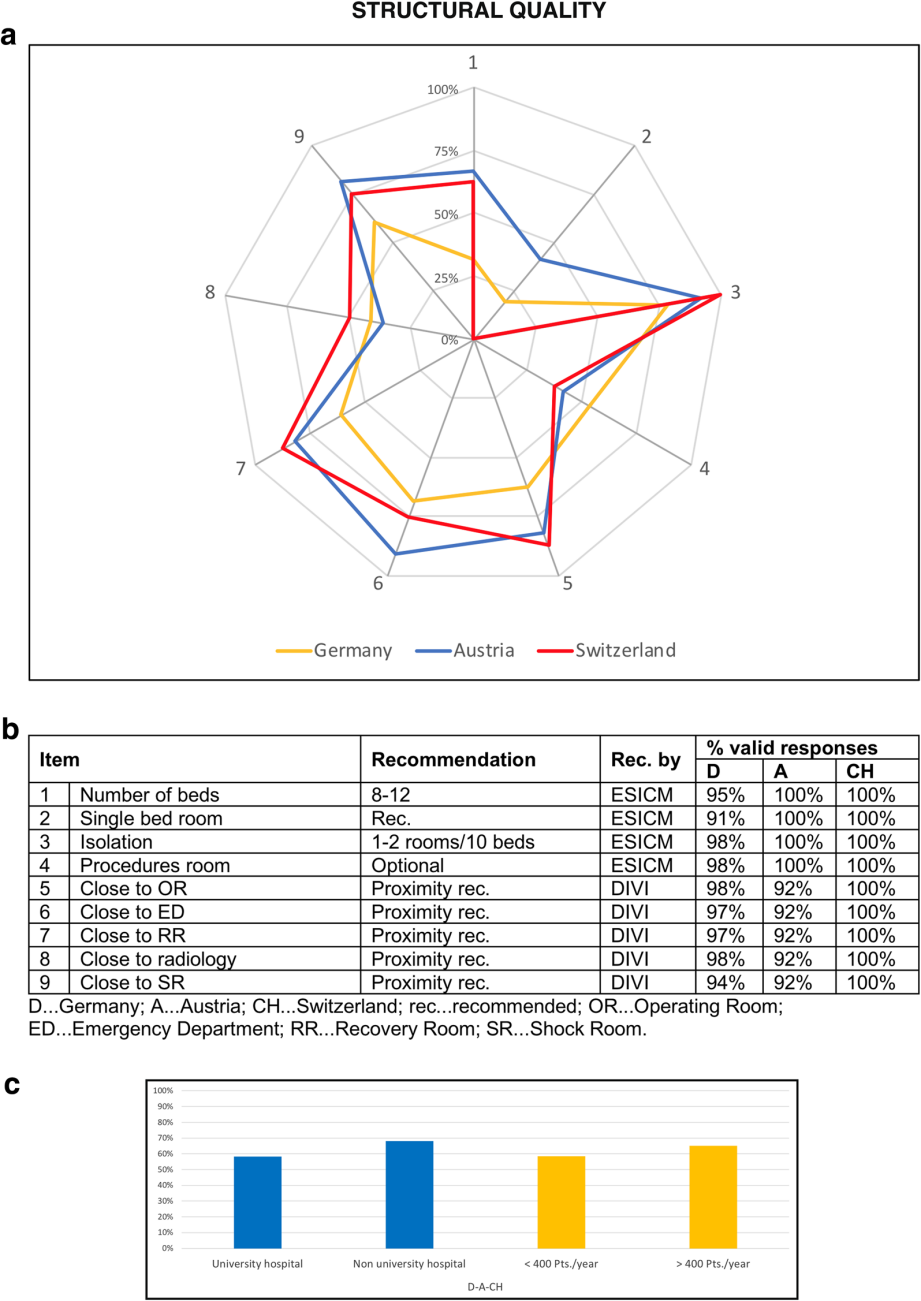
Structural quality of PICUs in the “D–A–CH” region. The web diagram (**a**) displays the percentage (of valid responses) of PICUs meeting the recommendations for the different items surveyed. In the table (**b**), the valid response rates for the different items are listed. The bar charts (**c**) display the mean of all nine items concerning structural quality according to the sub-categories university/non-university hospital and ≤ 400/> 400 patients treated annually

Overall structural recommendations were fulfilled by 53% of PICUs in Germany, 68% in Austria and 64% in Switzerland. Figure 1 gives a detailed overview of the nine structural items addressed in this survey. Criteria for structural quality were met to a higher percentage in non-university hospitals and of PICUs treating more than 400 patients per years when compared to university hospitals and PICUs treating less than 400 patients per years, respectively (Fig. 1c).

The median number of beds for all three countries was 12 ranging from 1 to 26 beds (median for D: 12; for A: 8; and for CH: 12). All PICUs in Switzerland had more than 12 beds and thus did not meet the ESICM recommendation of 8–12 beds. Not all wards could provide isolation rooms, but these were available in a large proportion of PICUs in Austria and Switzerland. Procedures rooms were documented in about 45% of all PICUs. Proximity to other vital Departments was defined as distance less than 3 min. In this regard, PICUs in Austria and Switzerland were predominantly located in a central position, while PICUs in Germany could grant the required proximity to the lowest proportion. A detailed overview of the different items according to the sub-categories, i.e. university and non-university hospitals and PICUs treating more and less than 400 patients annually is shown in Additional file 2.

### Diagnostic and therapeutic equipment

**Fig. 2:**
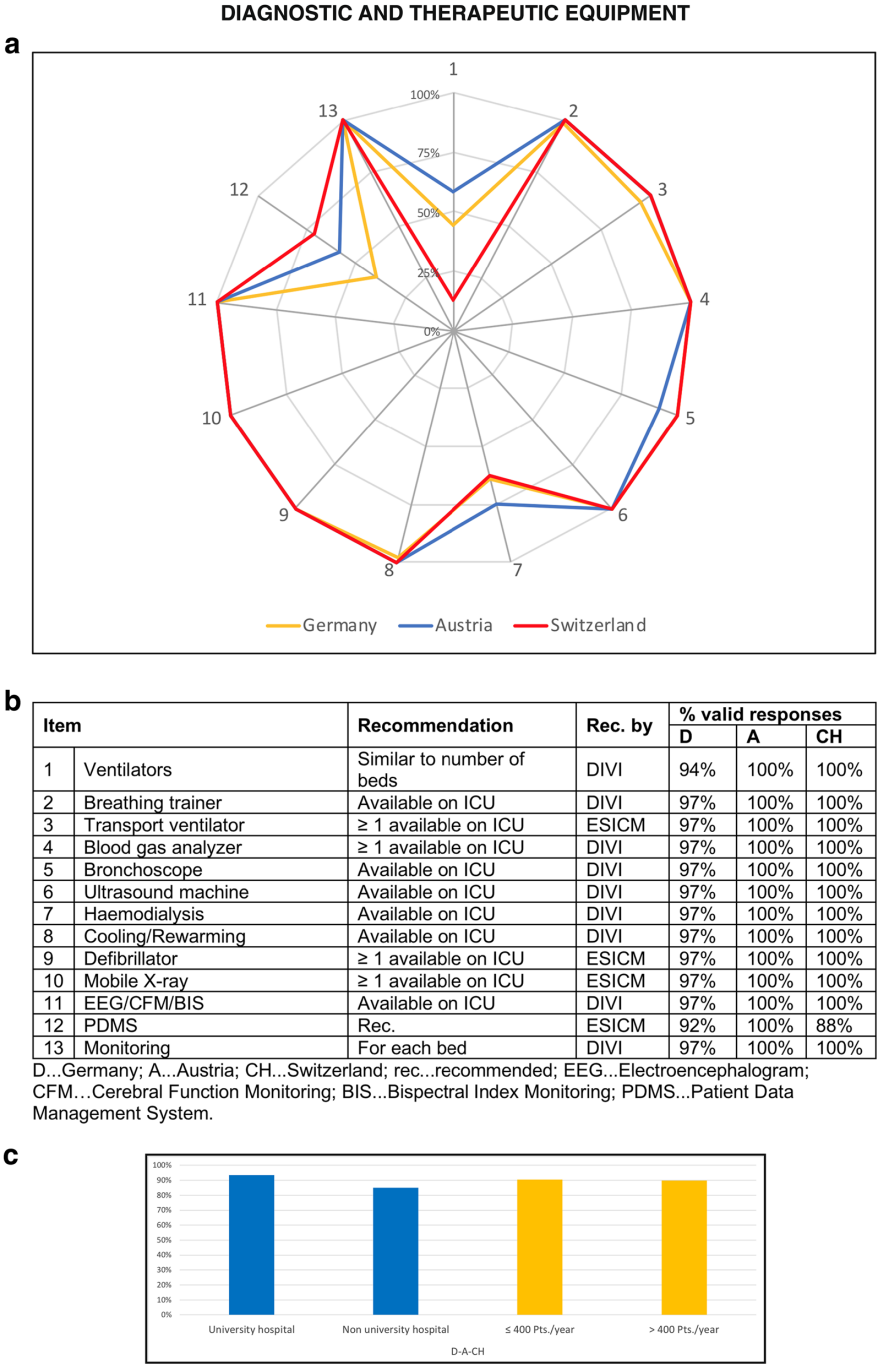
Diagnostic and therapeutic equipment of PICUs in the “D–A–CH” region. The web diagram (**a**) displays the percentage (of valid responses) of PICUs, which met the recommendations for the different items surveyed. In the table (**b**), the valid response rates for the different items are listed. The bar charts (**c**) display the mean of all 13 items concerning diagnostic and therapeutic equipment according to the sub-categories university/non-university hospital and ≤ 400/> 400 patients treated annually

Recommendations regarding diagnostic and therapeutic medical equipment were met by 88% of PICUs in Germany, 91% in Austria and 88% in Switzerland. Figure 2 depicts the respective 13 items of the questionnaire. University hospitals fulfilled the recommendations to a higher degree when compared to non-university hospitals. However, the number of annually treated patients did not influence the compliance with criteria regarding diagnostic and therapeutic equipment (Fig. 2c).

The recommendations for ventilators were met by less than 40% of all PICUs. The lowest results regarding the ventilator/bed rate were obtained in Switzerland followed by Germany and Austria. Additionally, not all PICUs were able to perform haemodialysis and not all of them had implemented a computer-based patient data management system (PDMS). A detailed sub-categorized overview of the different items regarding medical equipment is presented in Additional file 2.

### Personnel

**Fig. 3:**
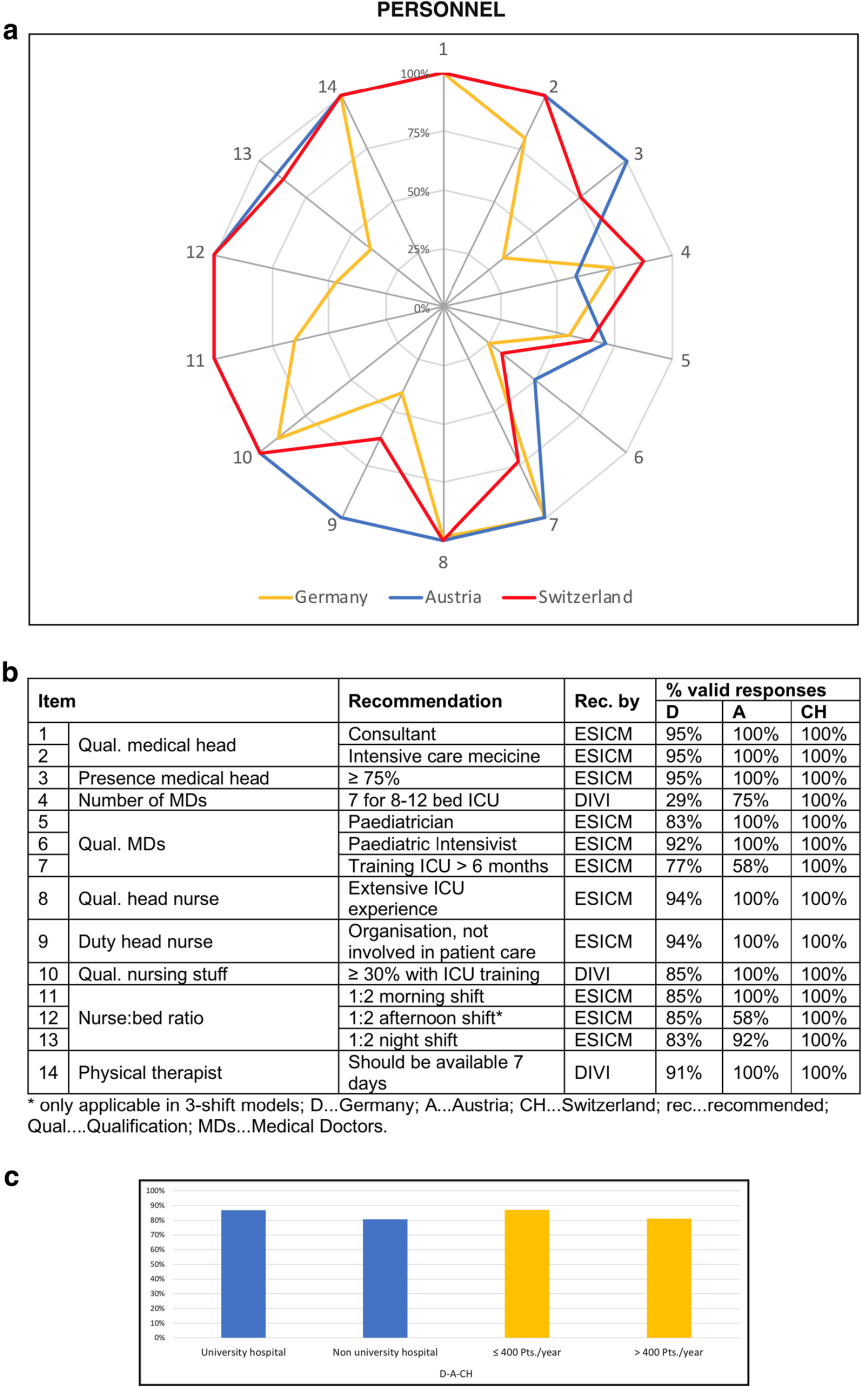
Personnel of PICUs in the “D–A–CH” region. The web diagram (**a**) displays the percentage (of valid responses) of PICUs meeting the recommendations for the different items surveyed. In the table (**b**), the valid response rates for the different items are listed. The bar charts (**c**) display the mean of all 14 items concerning personnel according to the sub-categories university/non-university hospital and ≤ 400/> 400 patients treated annually

PICUs were predominantly headed by male physicians in all three studied countries (D: 81%; A: 75%; and CH: 88%). The requirements regarding personnel were met by 71% of PICUs in Germany, 94% in Austria and 88% in Switzerland. Figure 3 shows the 14 items addressed in the questionnaire. University hospitals and PICUs treating less than 400 patients annually fulfilled the requirements to a slightly higher degree (Fig. 3c).

The heads of all PICUs were fully trained paediatricians (consultants). In contrast to Germany, all Austrian and Swiss heads had an additional specialization in paediatric intensive care medicine. Additionally, the presence of the head was lowest in Germany followed by Switzerland and Austria. A large proportion of the head nurses was routinely involved in patient care and could not solely concentrate on the organization of their ward and staff. The recommended nurse-to-bed ratio of 1:2 was met by all PICUs in Austria and Switzerland (during day time). In Germany, however, only a small proportion could grant this ratio in the morning shifts with a further decrease in nursing staff in the afternoon and night shifts.

A detailed sub-categorized overview of the 14 surveyed items regarding personnel is given in Additional file 2.

### Organization and quality improvement

**Fig. 4:**
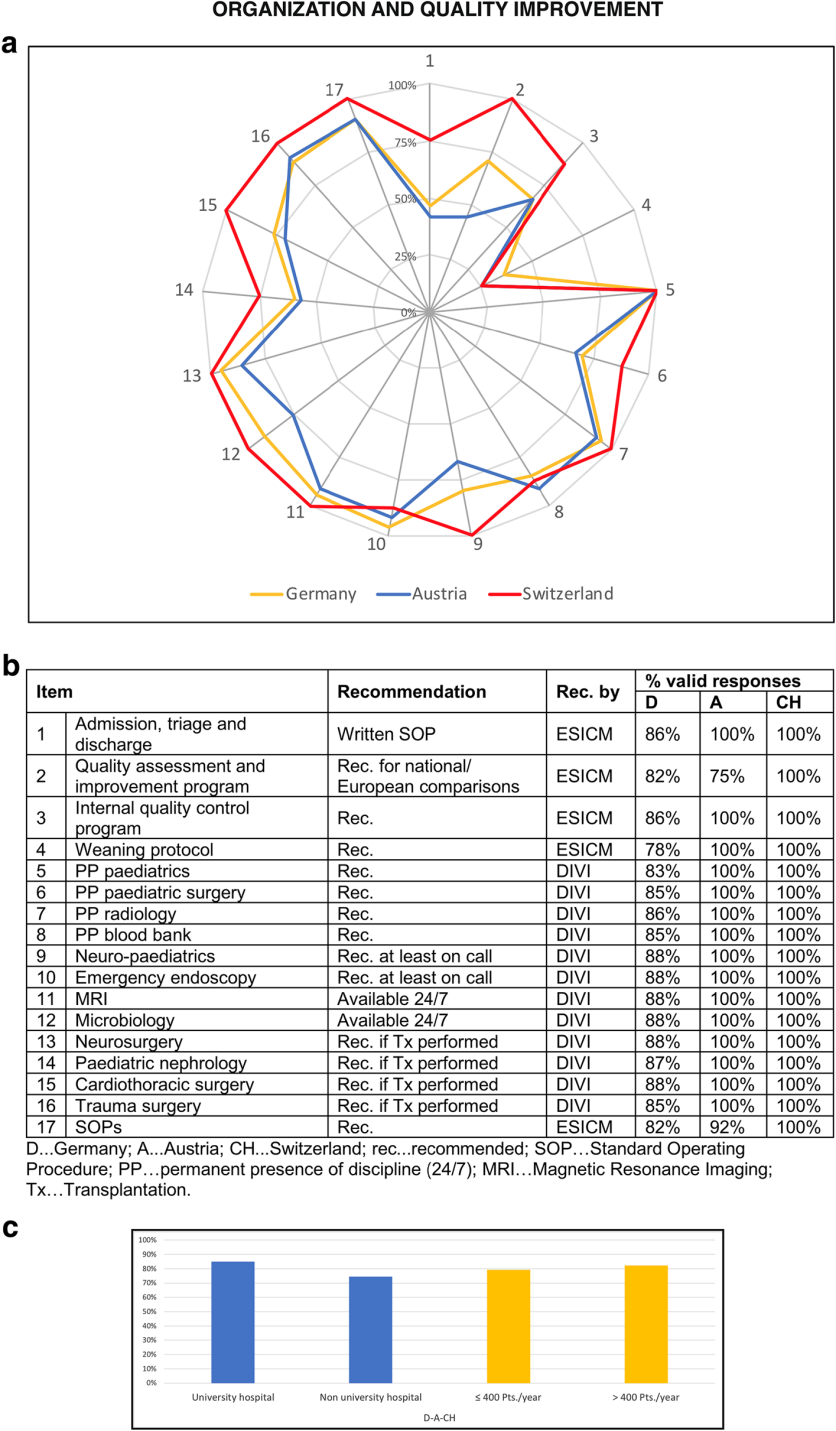
Organization and quality improvement in PICUs in the “D–A–CH” region. The web diagram (**a**) displays the percentage (of valid responses) of PICUs, meeting the recommendations for the different items surveyed. In the table (**b**), the valid response rates for the different items are listed. The bar charts (**c**) display the mean of all 17 items concerning organization and quality improvement according to the sub-categories university/non-university hospital and ≤ 400/> 400 patients treated annually

The guidelines for the organization of the PICUs were met in 79% in Germany, 74% in Austria and 90% in Switzerland. More detailed information about the 17 different items is shown in Fig. 4. The criteria regarding organization and quality improvement were best met by university hospitals (Fig. 4c).

Written standard operating procedures (SOPs) for admission, triage and discharge were provided by about half of participating PICUs in Germany and Austria with the best results obtained in Switzerland (75%). Most PICUs in Germany and Switzerland, but less than 50% in Austria, participated in benchmarking (quality assessment and improvement programme). Finally, weaning protocols were provided only by a small number of PICUs.

A detailed overview of the different items according to the sub-categories is shown in Additional file 2.

## Discussion

Results of the present survey offer up-to-date insights into the organization of PICUs in three central European countries. The focus on the paediatric setting and the combination of data derived from 85 PICUs of three European countries clearly distinguish this study from previously published reports which either were conducted on national levels or have focused on the adult population [19–21]. Based on the 2011 ESICM guidelines [11], our questionnaire has assessed infrastructural quality. In summary, the highest accordance with the guidelines was found for Switzerland followed by Austria and Germany. The high accordance of Swiss PICUs may be related to the fact that all Swiss ICUs are certified according to (adult-based) national guidelines closely related to those of the ESICM.

### Structural quality

Deficits regarding structural quality were found in all three studied countries but especially in Germany (compare Fig. 1). The most plausible reason seems to be the more centralized organization of paediatric intensive care in Switzerland when compared to the other two countries. Despite the fact that is been shown that the use of single-patient rather than multi-patient rooms improves nurses’ perceptions of the PICU environment for promoting patients’ sleep and the nurses’ own work experience [22], the recommended single and double bedrooms were available in only a low number of PICUs. A possible underlying reason may be the fact that most hospitals in the studied countries are based on older buildings with architectural limitations. Since isolation is a recognized preventive measure in infectious patients [23], this insufficiency needs to be addressed in the near future. PICUs were centrally located in about three quarters of hospitals. In our questionnaire, a 3-min walking distance was defined as proximity, because this seemed to be a reasonable distance in case of life-threatening emergencies to the authors. Nevertheless, there are no clear guidelines in this regard and the cut-off was arbitrary. In future, on-site visits could lead to an optimization in the evaluation of the wards’ location [24].

Comparing the three countries, Switzerland had the highest mean number of patients per year on PICUs. Astonishingly, the median stay on the PICU in Switzerland was about half that of Austria and Germany. This may be due to differences regarding the availability of alternatives such as IMCUs for less severe cases (who occupy PICU capacity). Additionally, Swiss hospitals provided the highest number of SOPs also including admission, triage and discharge (compare Fig. 4), which may have impact on the length of stay. On the other hand, there may be differences in the quality of aftercare (allowing earlier transfer to the normal ward to free PICU capacity) which is difficult to assess and was thus not evaluated in this survey.

### Diagnostic and therapeutic equipment

The diagnostic and therapeutic equipment was on a very high technical level in the majority of PICUs in all three studied countries (compare Fig. 2). However, our survey has revealed two obvious deficits: the availability of respirators and the implementation of a PDMS. Since the ESICM does not specify this item the requirement of one respirator per available bed was based on DIVI guidelines [12]. The reasonability of one respirator per bed on PICUs, however, can be discussed. The low number of PICUs using PDMS—especially in Germany—has to be criticized because PDMS has been shown to play a pivotal role in QM [15, 25].

### Personnel

Deficits regarding the personnel were encountered in all three surveyed countries (see Fig. 3). The training, qualification and presence of the medical head were insufficient in many German PICUs. With regard to patient safety and QM, a strict adherence to the guidelines has to be demanded [11–13, 26]. The low number of medical doctors (MDs) on the PICUs and their qualification mirrors tight resources in all countries. This fact cannot be overstressed since it has been shown that paediatric critical care provided in the PICUs staffed with a 24/7 intensivist presence is associated with improved overall patient survival [27]. Therefore, future training programmes should aim to raise the qualification of MDs on PICUs in order to further improve patient care [17].

The qualification of the nursing staff and the head nurse was very high, and a large proportion of PICUs met the requirements. However, the head nurse was also occupied with patient care in many wards, which contradicts the recommendations [11, 12]. The ratio between beds and nurses was tremendously different between the countries. While the requirements were met by the majority of PICUs in Austria and Switzerland, grave deficits were encountered in Germany where one nurse had to care for 3–5 critically ill children during the night shifts. Therefore, prompt measures have to be taken to improve this deficit [7, 11, 12, 23, 28–30].

### Organization and quality improvement

Concerning organization and quality improvement, Switzerland took a clear leading position in this survey (Fig. 4). In future, patient care and management could be improved by the increasing the number of available SOPs. In particular, the important role of a weaning protocol is well known [32] and the request for improved patient management warrants improvements in SOPs in all countries in this regard.

Moreover, it has previously been shown that both benchmarking and internal quality control improve patient safety and management [24, 31]. Although systems of quality comparison are available in Europe (i.e. Vermont Oxford Neonatal Networks, Extracorporeal Life Support Organization Registry), only half of the PICUs in Austria and Germany participate in these tools.

### Study limitations

The number of returned questionnaires was 100% for Austria and Switzerland. Thus, the results demonstrated above are representative for these countries. In contrary, the response rate for Germany was lower (61%). Nevertheless, the response rate of this survey is higher than that reported in previous smaller studies [19, 33].

A possible limitation of the present study is the fact that a survey is always based on the subjective opinion of the persons reporting for the centre. However, this “self-reporting” bias is the nature of a questionnaire-based survey. This could be mitigated by on-site visits from an independent committee. However, this was beyond the scope of this study.

Inclusion of a case mix index (CMI) would have further enhanced this study. The CMI was part of the questionnaire. However, response rates were very low (< 10%) and therefore not representative. All participating hospitals and the national societies were contacted a second time, but the response rate could not be increased. Therefore, this item had to be excluded in the final analysis. Additionally, the calculation of the CMI differs between European countries making a direct comparison difficult.

Another limitation lies in the interpretation of the guidelines. First of all, there are no available European guidelines specifically for paediatric patients. For a lack of alternatives, we had to rely on adult guidelines. Additionally, a similar approach has been chosen in a previous smaller survey [33]. The major difference between PICUs and adults ICUs can be found in the demands and (patho)physiology of the patients treated. Besides different requirements concerning medical equipment, the heterogenic spectrum within paediatrics causes difficulties in creating SOPs as well as guidelines. Thus, certain adult-based items may be the difficult to meet for PICUs.

Moreover, ESICM guidelines are based on treatment levels to a certain amount [10, 11]. The definition of these levels either is absent or differs between the countries making their comparability difficult. Additionally, ESICM guidelines are unspecific in certain items and had to be supplemented by DIVI recommendations [12] in certain questions to obtain a more complete image of the infrastructure of PICUs addressed in this study. DIVI guidelines were chosen because all countries were German speaking and the national societies of Austria and Switzerland have not published separate recommendations.

## Conclusion

While European recommendations regarding structural quality and equipment were met to a large proportion, deficits regarding personnel, organization and quality improvement were encountered. The present survey uncovers these deficits and discusses possible future interventions in order to further improve patient care and safety. Focusing on case mix index and outcome could enhance these data in future studies. Results of our study underline the need for organizational and process guidelines which are specific to paediatric intensive care units. Ideally, these should be based on evidence and implemented on a European level under control of the effect on quality and outcome.

## Additional files

**Additional file 1.** Questionnaire.

**Additional file 2.** Achieved items by subcategories.

## Abbreviations

D–A–CH: Germany–Austria–Switzerland
DIVI: Deutsche Interdisziplinäre Vereinigung für Intensiv- und Notfallmedizin
ESICM: European Society of Intensive Care Medicine
ICU: intensive care unit
IMCU: intermediate care unit
NICU: neonatal intensive care unit
PDMS: patient data management system
PICU: paediatric intensive care unit
QM: quality management
