# Supplementary material for: A multinational survey on the infrastructural quality of paediatric intensive care units
Source: Ann Intensive Care. 2018 Nov 6;8:105. doi: 10.1186/s13613-018-0451-1 (PMC6219992; doi:10.1186/s13613-018-0451-1)
Supplement: Supplementary file 1 — Additional file 1. Questionnaire. [file 13613_2018_451_MOESM1_ESM.pdf]

GENERAL INFORMATION ABOUT YOUR PAEDIATRIC INTENSIVE CARE UNIT (PICU)

1.) How many patients do you treat in your ICU ward annually?

N=…..

2.) What is the mean duration that patients stay on your ICU (in days)?

……days

3.) Does your centre perform organ transplantations?

□ yes
□ no

4.) How many per cent of the patients on your ICU ward could also be treated in an intermediate care unit (IMCU) annually?

……%

PART I – INFRASTRUCTURE

1.) **How many beds does your ICU have (number of beds)?**

N=.....

2.) **How are these beds distributed (insert number of each subcategory; if answers are not applicable please insert 0)?**

Rooms with 1 bed	N=.....
Rooms with 2 beds	N=.....
Rooms with 3 beds	N=.....
Rooms with 4 beds	N=.....
Other	N=.....

3.) **How many rooms of the following specifications do you have on your ICU ward? (number of rooms; if none please enter 0)**

Rooms to isolate immunosuppressed patients (rooms class I with patient sluice – sluice has under-pressure to patient room and overpressure to hall/corridor): N=…..

Rooms to isolate patients with communicable diseases (rooms class II with patient sluice – sluice has overpressure to patient room and under-pressure to hall/corridor; HEPA filter installed): N=…..

Isolation rooms (class I or II, sluice with under-pressure on both sides of the sluice; HEPA filter installed): N=…..

4.) **Do you have a designated procedures room for small surgical interventions?**

□ yes
□ no

5.) **How much time does it take to reach the operating room (theatre) of your department/hospital from your ICU (in minutes; if answers are not applicable in your centre please answer with N/A)?**

......min

6.) **How much time does it take to reach the emergency department of your department/hospital from your ICU (in minutes; if answers are not applicable in your centre please answer with N/A)?**

......min

7.) **How much time does it take to reach the recovery room of your department/hospital from your ICU (in minutes; if answers are not applicable in your centre please answer with N/A)?**

......min

8.) **How much time does it take to reach the radiology department of your department/hospital from your ICU (in minutes; if answers are not applicable in your centre please answer with N/A)?**

......min

9.) **How much time does it take to reach the shock room of your department/hospital from your ICU (in minutes; if answers are not applicable in your centre please answer with N/A)?**

......min

PART II – DIANGOSTIC AND THERAPEUTIC EQUIPMENT

Which of the following equipment is available on your ICU (please enter the number of available equipment)?

		N=
1.)	How many of your beds are equipped with an ICU ventilator?	
2.)	Breathing trainer(s)	
3.)	Transport ventilator(s)	
4.)	Blood gas analyzer(s)	
5.)	Bronchoscope(s)	
6.)	Ultrasound machine(s)	
7.)	Haemodialysis	
8.)	Cooling/Rewarming device(s)	
9.)	Defibrillator(s)	
10.)	Mobile X-ray	
11.)	EEG/CFM/BIS	
12.)	PDMS	
13.)	Monitoring device(s)	

PART III – PERSONNEL:

1.) **Which specialty or specialties (multiple answers allowed) is the head of your PICU ward trained in (please tick correct box(es))?**

□ Paediatrics
□ Paediatric Surgery
□ Anaesthesiology
□ Other

2.) **Does the head of your Department hold an additional diploma in intensive care medicine (please tick correct box)?**

□ yes
□ no

3.) **How much of his/her time (in percent) does the head of your PICU ward spend on patient care (please tick correct box)?**

□ approximately 25%
□ approximately 50%
□ approximately 75%
□ approximately 100%

4.) **How many medical doctors work on your PICU ward (including head and deputy)?**

N=…..

5.) **How many consultants do you employ on your PICU?**

N=…..

6.) **How many of your medical doctors have an additional diploma in intensive care medicine?**

N=…..

7.) **How many of your MDs have a training > 6 months in paediatric intensive care medicine?**

N=…..

8.) **Did the head nurse of your ICU ward receive special training in intensive care medicine?**

□ yes
□ no

9.) **Is the head nurse of your ICU ward also involved in patient treatment?**

□ yes
□ no

10.) **What is the percentage of nurses with intensive care training in relation to all nursing personnel of your PICU ward?**

……%

11.) **For how many patients is a nurse of your PICU ward responsible in the morning shift?**

N=…..

12.) **For how many patients is a nurse of your PICU ward responsible in the day shift?**

N=…..

13.) For how many patients is a nurse of your PICU ward responsible in the night shift?

N=…..

14.) Do you have a psychotherapist involved in patient care on your ICU ward?

□ yes
□ no

PART IV – ORGANIZATION AND QUALITY IMPROVEMENT

1.) **Do you have written SOPs for admission, transfer and discharge of ICU patients?**

□ yes
□ no

2.) **Does your ICU ward take part in external quality comparisons?**

□ yes
□ no

3.) **Do you provide an internal system for quality control/quality management on your ICU?**

□ yes
□ no

4.) **Do you apply a weaning protocol?**

□ yes
□ no

5.) **Does your centre offer permanent presence of a paediatric department**

□ yes
□ no

6.) **Does your centre offer permanent presence of a paediatric surgery department**

□ yes
□ no

7.) **Does your centre offer permanent presence of a radiology department?**

□ yes
□ no

8.) **Does your centre offer permanent presence of a blood bank?**

□ yes
□ no

9.) **Does your centre offer presence (at least on call) of a neuro-paediatrician?**

□ yes
□ no

10.) **Does your centre offer (at least on call) of an emergency endoscopy team?**

□ yes
□ no

11.) **Is an MRI available at your centre 24/7?**

□ yes
□ no

12.) **Is microbiology available at your centre 24/7?**

□ yes
□ no

13.) Does your centre offer permanent presence of a neurosurgeon?

□ yes
□ no

14.) Does your centre offer permanent presence of a paediatric nephrologist?

□ yes
□ no

15.) Does your centre offer permanent presence of a cardio-thoracic surgeon?

□ yes
□ no

16.) Does your centre offer permanent presence of a trauma surgeon?

□ yes
□ no

17.) Do you apply standard operating procedures (SOPs)?

□ yes
□ no
